# A comparative analysis of diagnostic values of high-frequency ultrasound and fiberoptic ductoscopy for pathologic nipple discharge

**DOI:** 10.1186/s12880-022-00885-4

**Published:** 2022-09-02

**Authors:** Hongmei Yuan, Xuemei Tang, Xurong Mou, Yuhong Fan, Xiang Yan, Jinsui Li, Lingmi Hou, Min Ren

**Affiliations:** 1 Department of Ultrasound, Sichuan Key Laboratory of Medical Imaging, Affiliated Hospital of North Sichuan Medical College, No. 1 MaoYuan South Road, Shunqing District, Nanchong, 637000 Sichuan China; 2 Department of Breast and Thyroid Surgery, Affiliated Hospital of North Sichuan Medical College, Nanchong, 637000 Sichuan China

**Keywords:** Fiberoptic ductoscopy, Ultrasound, Nipple discharge, Breast neoplasms, Diagnostic method of the breast

## Abstract

**Background:**

This study aimed to compare the diagnostic accuracy of high-frequency ultrasound (HFUS) and fiberoptic ductoscopy (FDS) for pathologic nipple discharge (PND).

**Methods:**

HFUS and FDS were conducted in 210 patients with PND (248 lesions) treated at our hospital. The diagnostic accuracy of these two methods was compared using pathological diagnosis as the standard.

**Results:**

Among 248 lesions, 16 and 15 of 16 malignant lesions were accurately diagnosed by HFUS and FDS, respectively. Of 232 benign lesions, 183 and 196 cases were accurately diagnosed by HFUS and FDS, respectively. The sensitivity, specificity, positive predictive value (PPV), and negative predictive value (NPV) of HFUS in diagnosis of intraductal lesions were 84.36% (95% CI 79.26–88.39%), 60% (95% CI 23.07–92.89%), 96.03% (95% CI 96.55–99.83%), and 7.31% (95% CI 2.52–19.4%) respectively. The sensitivity, specificity, PPV, and NPV of FDS in diagnosis of intraductal lesions were 86.83% (95% CI 82.00–90.52%), 100% (95% CI 56.55–100%), 100% (95% CI 98.21–100%), and 13.51% (95% CI 5.91–27.98%) respectively. Diagnostic accuracy rates of HFUS and FDS were 83.87% (208/248) and 85.08% (211/248), respectively, exhibiting no statistically differences (χ^2^ = 0.80, *P* > 0.05). The accuracy of HFUS combined with FDS was 93.14% (231/248), showing statistically differences (χ^2^ = 10.91, *P* < 0.05).

**Conclusions:**

Both HFUS and FDS demonstrated high diagnostic values for PND. HFUS has the advantage of non-invasive for nipple discharge with duct ectasia, exhibited good qualitative and localization diagnostic values. It is the preferred evaluation method for patients with nipple discharge. When HFUS cannot identify the cause of PND, FDS can be considered.

## Background

Pathologic nipple discharge (PND) is a unilateral, spontaneous, sanguineous, or serous discharge. It is the third most common symptom besides breast pain and lumps [1]. Common etiologies of PND include intraductal papilloma, duct ectasia, and fibrocystic changes, with a malignancy rate of approximately 5–15% [2, 3]. Using a conventional “one-size-fits-all” breast duct excision to rule out malignancy in patients with PND is inappropriate [4, 5]. Therefore, qualitative and localization diagnoses are essential for PND. PND evaluation methods include discharge smears, galactography [6], enhanced magnetic resonance imaging (MRI) [7, 8], fiberoptic ductoscopy (FDS) [9], and ultrasonography (US) [10]. FDS have direct visualization and high diagnostic value for intraductal lesions [9], while it is an invasive and time-consuming method. In recent years, improvement in HFUS resolution has led to this technique's application in breast duct examination. This convenient, radiation-free method is readily accepted by patients [11, 12]. However, only a few reports have compared diagnostic values of HFUS and FDS for PND. Thus, this paper aimed to compare diagnostic values of HFUS and FDS for PND to provide a sound rationale for clinical selection of reasonable treatment regimens.

## Methods

### Subjects

This was a retrospective study of the patients with PND who underwent HFUS and FDS at our hospital between July 2013 and May 2021, excluding the cases of physiological nipple discharge during pregnancy and lactation. All lesions were pathologically confirmed.

### Instruments and methods

For HFUS, high-frequency color Doppler ultrasound diagnostic machines were used, including Philips IU22, GE S9, Mindray Resona7, and Canon Aplio 500. Accordingly, the probe frequency was set to be 5–10 MHz in all ultrasound instruments. The patients lay supine and raised both arms to fully expose breasts and bilateral axillary fossa. A lateral decubitus position was added if necessary. Bilateral breasts were inspected comprehensively to examine the incidence of ductal widening, smoothness of ductal walls, and presence of space-occupying lesions in the ductal lumen. The relationship between the lesion and the duct, lesion morphology, margins, internal echoes, blood flow characteristics, and changes in surrounding tissues were determined.

For FDS, FVS-6000MI endoscopic imaging light source system was used. The patient lay in the supine position. After routine disinfection, the pressure was applied to the discharging nipple to locate the discharging duct orifice. After this, 2% lidocaine was injected into the duct orifice. Bowman lacrimal probes dilate the orifice before the FDS was inserted gradually. Various hierarchical branches of the discharging lactiferous duct were examined one by one to inspect the ductal wall structure and the presence of space-occupying lesions in the lumen. Sites and characteristics of lesions were recorded. Moreover, lesions' morphology, size, color, and activity of lesions were assessed.

For pathology, ultrasound guided core biopsy, local resection, or surgical resection of the lesion was performed, and then the pathological results were obtained.

### Statistical analysis

All statistical analyses were performed using SPSS 22.0 (IBM Corp., Armonk, NY, USA). Fisher’s exact test and χ^2^ test was used for the statistical analysis, 95% confidence intervals (95% CI) of sensitivity, specificity, positive predictive value (PPV), and negative predictive value (NPV) of HFUS and FDS were calculated. *P* < 0.05 was considered significant.

## Results

The study involved 210 patients with PND who underwent HFUS and FDS examinations. All patients were females. Their mean age was 48.0 ± 4.6 years (16–72 years). We observed 248 lesions, including 172 unilateral and 38 bilateral lesions. Lumps were palpable in 32 lesions. The mean course of the disease was 7 ± 6.3 months (1 day–4 years).

### HFUS and FDS examination results

High-frequency HFUS, FDA, and pathological results of 248 cases of PND are summarized in Table 1. Of 248 lesions, 16 were malignant, all of which were accurately diagnosed by US, whereas FDS missed one. Overall, 232 were benign lesions, including 163, 35, 9, 12, 8, and 5 cases of intraductal papilloma, adenosis with duct ectasia, apocrine adenosis with usual ductal hyperplasia, usual ductal hyperplasia with inflammatory cell infiltration, fibrocystic changes with ductal hyperplasia, and mammary duct ectasia, respectively. The sensitivity, specificity, PPV, and NPV of HFUS in the diagnosis of intraductal lesions were 84.36% (95% CI 79.26–88.39%), 60% (95% CI 23.07–92.89%), 96.03% (95% CI 96.55–99.83%), and 7.31% (95% CI 2.52–19.4%) respectively. The sensitivity, specificity, PPV, and NPV of FDS in the diagnosis of intraductal lesions were 86.83% (95% CI 82.00–90.52%), 100% (95% CI 56.55–100%), 100% (95% CI 98.21–100%), and 13.51% (95% CI 5.91–27.98%) respectively. For the benign lesions, 192 and 196 were accurately diagnosed by HFUS and FDS. Overall diagnostic accuracy rates of HFUS and FDS were 83.87% (208/248) and 85.08% (211/248), respectively, showing no statistically significant difference between the two (χ^2^ = 0.80, *P* > 0.05). When pathological diagnoses were used as the standard, 204 lesions were accurately diagnosed by HFUS and FDS. Overall, 39 cases accurately diagnosed by HFUS were missed or misdiagnosed by FDS, meanwhile, 36 cases accurately diagnosed by FDS were missed or misdiagnosed by HFUS. However, HFUS combined with FDA accurately diagnosed 231 lesions, missing or misdiagnosing only 17. This, yielded a diagnostic accuracy of 93.14% (231/248), which was significantly higher than either HFUS or FDS alone (χ^2^ = 10.91, *P* < 0.05).

**Table 1:** Diagnostic accuracy rates [% (lesion)] of high-frequency HFUS and FDS

Pathological results	Examination methods	
	HFUS	FDS
Benign	82.75% (192/232)	84.48% (196/232)
Intraductal papilloma (n = 163)	87.12% (142/163)	89.57% (146/163)
Adenosis with duct ectasia (n = 35)	71.43% (25/35)	65.71% (23/35)
Apocrine adenosis with usual ductal hyperplasia (n = 9)	88.89% (8/9)	77.78% (7/9)
Usual ductal hyperplasia with inflammatory cell infiltration (n = 12)	50% (6/12)	83.33% (10/12)
Fibrocystic changes with ductal hyperplasia (n = 8)	100% (8/8)	62.5% (5/8)
Mammary duct ectasia (n = 5)	60% (3/5)	100% (5/5)
Malignant	100% (16/16)	93.75% (15/16)
Ductal carcinoma in situ (DCIS) (n = 12)	100% (12/12)	100% (12/12)
Invasive breast carcinoma of no special type (n = 4)	100% (4/4)	75% (3/4)

### High-frequency HFUS and FDS imaging features

#### Benign intraductal space-occupying lesions

**Fig. 1:**
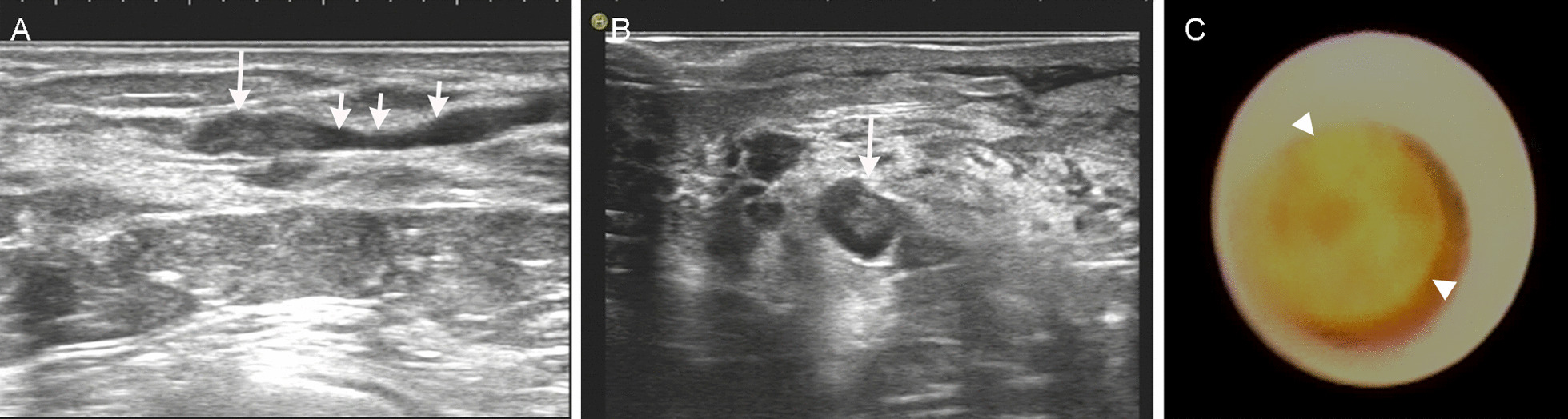
A 45-year-old female patient with yellow nipple discharge for 1 month. Comparison of ultrasound sonogram and fiberoptic ductoscopy for intraductal papilloma was performed. Sections from **A** the long and **B** the short axes in conventional two-dimensional ultrasound revealed local ductal dilation (short arrow) in the 3 o'clock direction in the right breast, with solid isoechoic nodular filling (long arrow) in the distal lumen of the dilated segment. Fiberoptic ductoscopy **C** showing dilation of the breast duct with smooth ductal walls and one light yellow wart-like neoplasm obstructing the lumen

In HFUS, intraductal papillomas (163 cases) included 121 cases of solitary type and 42 cases of multiple type. Solitary intraductal papillomas (121 cases) were shown as hypoechoic and isoechoic solid nodules in dilated ducts (n = 73), solid nodules at the end of dilated ducts (n = 21; Fig. 1A, B), nodules without duct dilation (n = 14), stripy duct dilation in the breast without space-occupying lesions (n = 8), or no abnormalities (n = 5). In FDS, Solitary intraductal papillomas were shown as milky-white or light yellow intraductal neoplasms with smooth surfaces (Fig. 1C), exhibiting smooth adjacent ductal walls, whereas duct dilation without space-occupying lesion was observed in 5 cases. For 42 cases of multiple intraductal papilloma, HFUS exhibited multiple small bulging lesions with smooth surfaces in tertiary or above dilated ducts, punctate blood flow signals were observed in the HFUS (n = 34). Localized simple ductal dilation was observed in 5 cases. However, 3 cases exhibited no significant abnormality. FDS revealed multiple wide basal bulging lesions in tertiary or quaternary lactiferous ducts or above, whereas in 12 lesions that FDS did not detect, duct dilation alone was observed without signs of intraductal lesions. For 35 cases of adenosis with duct ectasia, HFUS revealed solitary hypoechoic nodules (n = 17), two or more hypoechoic nodules (n = 7), or one solitary hyperechoic nodule (n = 1) in dilated ducts. FDS revealed solitary (n = 16) or multiple (n = 7) intraductal neoplasms. Neoplasms were mostly light yellow (n = 18), with some being milky white (n = 5). However, for the remaining 12 cases, lesions were not detected by FDS. For 9 cases of apocrine adenosis with usual ductal hyperplasia, HFUS revealed duct dilation with cystic solid nodules (n = 6), hypoechoic nodules within dilated ducts (n = 2), or duct dilation alone (n = 1). However, FDS revealed intraductal neoplasms (n = 6) or spiculated ductal wall (n = 1). Lesions were not detected in 2 cases.

#### Malignant intraductal space-occupying lesions

**Fig. 2:**
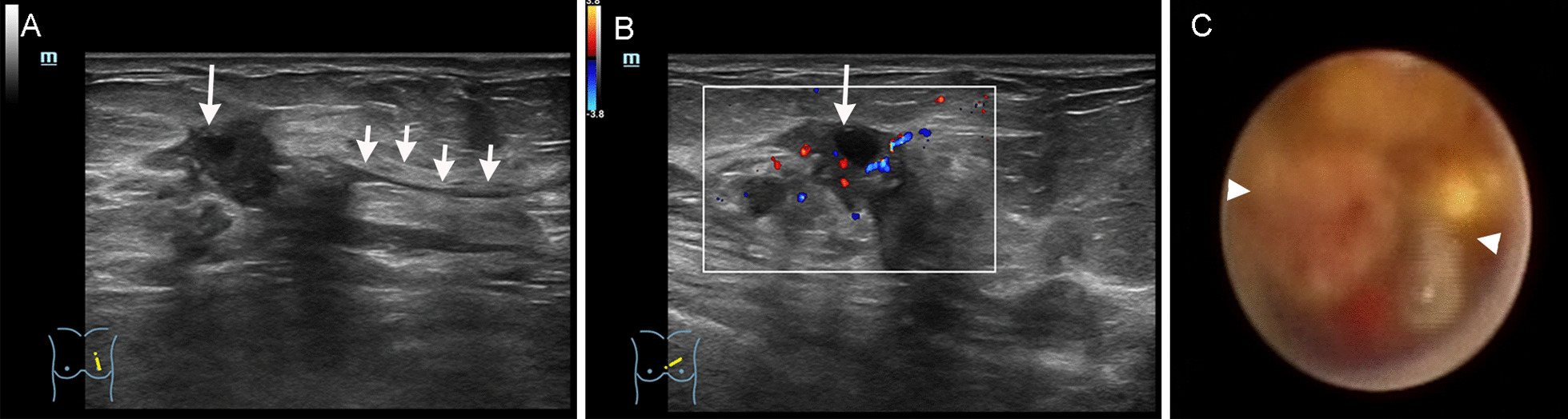
A 70-year-old female patient with bloody nipple discharge for 3 months. Comparison of ultrasound sonogram and fiberoptic ductoscopy of invasive ductal carcinoma of the breast was performed. Conventional two-dimensional ultrasound **A** showing a 2.2 cm × 1.2 cm cystic solid nodule (long arrow) in the gland layer approximately about 3 cm from the nipple in the 10 o'clock direction in the left breast. The nodule morphology was irregular, exhibiting “crab feet”-like margins. Adjacent duct dilation (short arrow) was observed, which extended to the nipple, showing poor intraductal sound penetration; CDFI (**B**) revealing punctate and short bar-like blood flow signals in the space-occupying lesion. FDS (**C**) revealing an irregular intraductal neoplasm completely obstructing the lumen, which bled easily when touched. Adjacent ductal walls were stiff with poor elasticity. The rear lumen was inaccessible

There were 16 malignant space-occupying lesions, including in situ breast ductal carcinoma (n = 12) and invasive ductal carcinoma (n = 4). HFUS revealed solid masses in dilated ducts (n = 6) and solid masses or cystic solid masses that extended from dilated ducts (n = 10; Fig. 2A), with marked blood flow signals detected in some masses (Fig. 2B). FDS revealed irregular neoplasms within the lactiferous duct (n = 15; Fig. 2C). However, one case was misdiagnosed as mammary duct infection because no mass was observed in the local luminal stenosis resulting from large quantities of white flocculation floating in the lumen.

#### Mammary duct ectasia, fibrocystic changes, and usual ductal hyperplasia with inflammatory cell infiltration

For 12 cases of usual ductal hyperplasia with inflammatory cell infiltration, FDS diagnosed 10, showing spiculated ductal walls in dilated ducts. Poor sound penetration in the ductal lumen was reflected by flocculant HFUS flotation and flow. HFUS revealed spiculated ductal walls in dilated ducts for 6 lesions. For 8 cases of fibrocystic changes, HFUS revealed cystic anechoic regions in all 8, of which 6 cystic anechoic regions were extended from dilated ducts. However, FDS only diagnosed 5, missing 3 because FDS could not reach proliferative cystic nodules located at the edge of the gland because of the scope diameter and length limitations. For 5 cases of mammary duct ectasia, 3 were correctly identified by HFUS whereas FDS correctly diagnosed all.

## Discussion

Nipple discharge may arise from various physiological and pathological causes. PND might be caused by breast lesions and factors extrinsic to the breast. The most common causes related to the breast are primarily benign, such as intraductal papilloma, adenoma, and fibrocystic changes. Malignant lesions account for less than 15% of PND [2, 3]. In this study, intraductal papilloma accounted for 65.73% (163/248), whereas breast cancer accounted for 6.45% (16/248), indicating the critical importance of accurately identifying the disease etiology. The World Health Organization stated that benign intraductal papillary lesions are associated with a 2_7.5 fold increase in the risk of subsequent invasive breast carcinoma [13]; The low malignant rate of intraductal lesions in this study may be related to the improvement of imaging diagnosis in recent years, leading to the early detection of some benign papillary lesions and treated timely treatment.

FDS can visualize the lesioned lactiferous duct directly with its endoscopic imaging light source system, enabling direct inspection of minimal lesions in the breast duct [14]. The sensitivity and specificity of FDS in this study was 86.83% and 100% respectively, whereas the literature reports were 53.2–99.88% and 60–99.33%, respectively [15, 16]. The difference between studies may be related to the type and location of cases included in the literature, such as the diagnostic efficiency of FDS for central (solitary) lesions is significantly higher than that of peripheral (multiple) lesions. Because the FDS scope has a semi-rigid cannula with a complicated operation. In addition, the nipple must present discharge before ducts can be entered for FDS examination. The breast tissue consists of 15–20 duct systems. Only after locating the discharging orifice can 1–2 duct systems be targeted for inspection. Only the ductal lumen can be observed, whereas its relationship with surrounding tissues cannot be visualized with an FDS scope. Furthermore, the observation range of FDS is limited by the scope's diameter, length, and curvature, making it impossible to reach terminal duct openings that are smaller than the scope diameter [16].

HFUS is a radiation-free, non-invasive, inexpensive, painless, and repeatable method, it can detect intraductal lesions with an inner diameter greater than 1 mm, the intraductal lesions could be more easily visualized since ducts have dilation effusion. The sensitivity and specificity of HFUS for PND in this study were 84.36% and 60% respectively, however, the previous literature reported 15–100% and 31–99.6% [12]. Difference of diagnostic efficacy may be related to the type of cases included and resolution of ultrasonic instrument in the different literatures. The diagnostic accuracy of HFUS was 87.12% for 163 cases of intraductal papilloma in our study, which was lower than that of FDS (89.57%), 5 cases of mammary duct ectasia were diagnosed by FDS but 2 cases were missed by HFUS, whereas Zhou have reported that the sensitivity of HFUS for mammary duct ectasia was 100% [17]. For the clinical examination may empty the intraductal fluid by applying pressure before HFUS examination, which might have led to false negatives. Meanwhile, the patients were asked not to apply pressure to the breast before FDS, which might have resulted in a higher positive rate of FDS than HFUS. Furthermore, some lesions exhibited nipple discharge in the early stage without marked duct dilation, which might have increased the propensity of missed diagnosis. The detection rates of HFUS and FDS were 80.95% (34/42), 71.42% (30/42) respectively for multiple intraductal papilloma in this study, HFUS was higher than FDS. This might be FDS localization and qualitative evaluation of the lesion were affected by factors such as limitations of FDS scope diameter and the technique of the operator [15]. Multiple intraductal papilloma is a proliferative change that occurs primarily in epithelial cells and the stroma of small ducts and terminal ducts, exhibiting a high rate of carcinomatous transformation. FDS often cannot reach these regions due to diameter limitations. In comparison, the accuracy of HFUS diagnosis was higher than the reported accuracy (75%) in previous studies [16], which is partly related to the enhanced resolution of HFUS instruments. Despite this, missed diagnoses still occur due to section thickness artifacts in HFUS, mainly when echoes of intraductal lesions are similar to those of surrounding breast tissues. In this study, all malignant intraductal lesions were correctly diagnosed by HFUS because significant masses had already formed. On the other hand, although FDS diagnosed 15 cases, it could only discover the presence of lesions. Indication of lesion extent and location in relation to surrounding tissues by FDS were inferior to those revealed by HFUS.

HFUS and FDS have advantages and disadvantages for diagnosing PND. HFUS is related to subjective judgment and experience of the operator, however, it is a non-invasive examination that is easily accepted by patients, making it highly valuable in clinical applications. To further improve diagnostic accuracy and decrease missed or misdiagnosis of HFUS, US examination of nipple discharge should be performed gently to reduce the pressure of ultrasonic probe on the breast. Upon discovery of a dilated breast duct, the dilated duct should be visualized as much as possible along the direction of the duct. When an intraductal lesion is found, multi-sectional scanning of the lesion should be performed to enable dynamic observation, meanwhile discerning the intraductal lesion and normal periductal structures. After identifying the intraductal lesion, the benign or malignant nature of the lesion should be determined based on its morphology, relationship with surrounding structures, and blood flow signals. For patients whose lesions cannot be revealed by HFUS, combining HFUS with FDS can significantly increase the diagnostic accuracy.

## Conclusion

In summary, HFUS of breast is a preferred evaluation method for patients with nipple discharge. If the cause of the disease, location of the lesion, and extent of the lesion can be established with HFUS, FDS examination is unnecessary. However, FDS can be considered to reduce missed diagnoses for patients with negative HFUS findings.

## Data Availability

All data generated or analysed during this study are included in this published article.

## Abbreviations

PND: Pathologic nipple discharge
FDS: Fiberoptic ductoscopy
HFUS: High-frequency ultrasound
MRI: Magnetic resonance imaging
